# Effect of Body Position on the Development of Intra-abdominal Hypertension and Abdominal Compartment Syndrome in Patients in Critical Condition

**DOI:** 10.7759/cureus.93192

**Published:** 2025-09-25

**Authors:** Maria Kirketsou, Georgia Toulia, Dimitrios Papageorgiou, Maria Polikandrioti, Niki Pavlatou, Antonia Kalogianni

**Affiliations:** 1 Nursing, University of West Attica, Athens, GRC; 2 Pathological and Critical Care Nursing, University of West Attica, Athens, GRC

**Keywords:** abdominal compartment syndrome (acs), head of bed elevation, intra-abdominal hypertension, intra-abdominal pressure measurement, mechanically ventilated patients

## Abstract

Background

Measurement of intra-abdominal pressure (IAP) in the supine position (SP-0^o^) is the cornerstone for preventing intra-abdominal hypertension (IAH) and abdominal compartment syndrome (ACS). However, mechanically ventilated patients (MVP) are nursed with a head-of-bed angle (HOB) at 30^o^-45^o^ for ventilator-associated pneumonia (VAP) prevention. The aim of the study was to evaluate the impact of HOB positioning of MVP on IAH and ACS.

Methods

A prospective observational cohort study was conducted at 150 MVP who had completed 24 hours with at least one risk factor for IAH in the mixed medical and surgical Intensive Care Unit (ICU). IAP, abdominal perfusion pressure (APP), and filtration gradient (FG) were measured via the bladder at SP-0^o^, 20°, 30°, and 45° HOB three times daily for 3 days.

Results

Mean Body Mass Index (BMI) was 30.4 (SD=6.4), while pulmonary infection was the leading admission cause. Out of 1800 registrations, IAP was reported 757 times (42.1%) and ACS 51 times (2.8%). Fourteen patients developed ΙΑΗ. IAH and ACS percentages differed significantly among the four body positions, with the lowest percentage being in SP-0^o^ and the highest at 45°. APP and FG in SP-0^o^ were significantly higher compared to 20^o^, 30^o^, and 45^o^ (p<0.001 for all comparisons). Greater BMI (OR=1.14; p<0.001) at 20^o^, 30^o^, and 45^o^ of HOB elevation was significantly associated with a greater risk of IAH than SP-0^o^.

Conclusion

Elevation of HOB increases IAP and risk of IAH. It is recommended that MVPs with increased BMI and with at least one risk factor for IAH have their IAP measured daily.

## Introduction

Intra-abdominal hypertension (IAH) and abdominal compartment syndrome (ACS) are associated with significant morbidity and mortality. Increased intra-abdominal pressure can reduce the abdominal perfusion pressure (APP) and negatively affect the perfusion of intra-abdominal organs [1], such as the kidneys, intestine, and liver, leading to organ failure and death. The prevalence of IAH and ACS ranges from 30% to 49% and 2%-32% respectively, while the mortality ranges from 5.8%-54% and 0%-100% [2]. The clinical development of IAH is a silent process, and it is not diagnosed until its progress has been completed. Measuring intra-abdominal pressure (IAP) is the cornerstone of preventing ACS. IAH is a sustained or repeated pathological elevation of IAP of 12 mmHg or greater. The World Society of Abdominal Compartment Syndrome (WSACS) has developed the grades of IAH (Stage I (12-15 mmHg), Stage II (16-20 mmHg), Stage III (21-25 mmHg), Stage IV (>25 mmHg)) [3].

The WSACS defines normal IAP as 0-5 mmHg in healthy people and 5-7 mmHg in critically ill adults, and recommends the measurement of IAP in patients at risk of IAH or ACS in the supine position via the bladder.

Most patients in the Intensive Care Unit (ICU) are mechanically ventilated and, for prevention of ventilator-associated pneumonia (VAP), are nursed with the head of bed (HOB) elevated to 30o-45o [4,5]. If the abdomen behaves as a liquid-filled container, according to Pascal’s law, the pressure remains constant regardless of body position since fluid is not compressible. Therefore, intra-abdominal pressure measurements can vary with body position, noticing higher IAP in semi-recumbent versus supine positions [6]. A few studies have quantified the relationship between specific HOB angles and IAH risk in a large cohort of mechanically ventilated ICU patients [7-11], and the question of whether ventilated patients in a semi-recumbent position are at risk of development of IAH or ACS remains unanswered.

## Materials and methods

Study design and setting

A prospective observational cohort study was conducted at a medical and surgical ICU of General Hospital of Athens for the period 2020-2023.

Participants

The study population was 150 mechanically ventilated patients over 18 years old who had completed 24 hours of hospitalization in the ICU with at least one risk factor for the development of intra-abdominal hypertension (Table 1).

**Table 1:** Risk factors for IAH and ACS [3] The permission has been obtained from the original publishers. IAH: intra-abdominal hypertension; ACS: abdominal compartment syndrome; APACHE: Acute Physiology and Chronic Health Evaluation score; SOFA: Sequential Organ Failure Assessment score; PEEP: Positive End-Expiratory Pressure

Diminished wall compliance	Increased intra-luminal contents
Abdominal surgery	Gastroparesis/gastric distention/ileus
Major trauma/ Major burns	Colonic pseudo-obstruction
Prone positioning	Volvulus
Capillary leak/fluid resuscitation	Others/ miscellaneous
Acidosis	Age
Damage control laparotomy	Bacteremia
Hypothermia	Coagulopathy
Increased APACHE-II or SOFA score	Increased head of bed angle
Massive fluid resuscitation or positive fluid balance	Massive incisional hernia repair
Polytransfusion	Mechanical ventilation
Increased intra-abdominal contents	Shock or Hypotension
Peritoneal dialysis	Obesity or increased body mass index
Acute pancreatitis	PEEP
Distended abdomen	Peritonitis
Hemoperitoneum/pneumoperitoneum or intra-peritoneal fluid	Pneumonia
Collections	Sepsis
Intra-abdominal infection/abscess	
Intra-abdominal or retroperitoneal tumors	
Laparoscopy with excessive insufflation pressures	
Liver dysfunction/cirrhosis with ascites	

Exclusion criteria

The exclusion criteria were: inability to change positions in bed due to underlying disease (e.g., spinal injuries), manipulation of the bladder (bladder surgery or injury), or severe hemodynamic instability.

Study protocol

Demographic, clinical, and laboratory data of the patients, including age, gender, Body Mass Index (BMI), disease diagnosis and severity (Acute Physiology and Chronic Health Evaluation II score (APACHE II), Sequential Organ Failure Assessment score (SOFA)), and length of stay, were recorded at baseline on a specially constructed form. Biochemical and specific markers of renal and hepatic function were measured twice - at baseline and after the end of all IAP measurements (day 0 and day 4). IAP, mean arterial pressure (MAP), abdominal perfusion pressure (APP), and filtration gradient (FG) were measured in every position. IAH was categorized into four stages according to WSACS. ACS is defined as a sustained IAP ≥20 mmHg, which is associated with new organ dysfunction. The IAP measurements were performed by ICU nurses.

Methods of IAP measurements

The IAP measurement was performed via a bladder Foley catheter according to the recommendations of WSACS [7]. The first measure of IAP was in SP-0°, with a transducer attached to the bladder catheter zeroed at the mid-axillary line. The IAP was expressed in mmHg and was measured at end-expiration, 30-60 seconds after instillation of 25 ml normal saline, to allow bladder detrusor muscle relaxation and after ensuring that abdominal muscle contractions were absent. The measurement procedure was carried out in the supine position (0°), at 20°, 30°, and 45° HOB, every 8 hours daily for up to three days. The instilled saline was completely drained from the bladder before the next measurement. The IAP was measured twice at each body position, in a 3-minute space, and the mean of the two measurements was calculated. Α total of 36 measurements were recorded for each patient.

## Results

The sample consisted of 150 patients, with a mean age of 63.1 years (SD=16.1 years). All patients were mechanically ventilated, with a mean positive end-expiratory pressure (PEEP) of 7.7 cmH2O and had at least one risk factor for IAH and ACS, with pneumonia (60.7%) and hypothermia (44%) being the most common. Sample characteristics are presented in Table 2.

**Table 2:** Sample characteristics (n=150) n: Number of participants; APACHE: Acute Physiology and Chronic Health Evaluation score; SOFA: Sequential Organ Failure Assessment score; FiO2: fraction of inspired oxygen

Sample characteristics	n (%)
Gender	
Male	89 (59.3)
Female	61 (40.7)
BMI levels	
Overweight	54 (36.0)
Obese	69 (46.0)
Reason for ICU admission	
Respiratory infection	39 (26.0)
SARS-COVID-19	56 (37.3)
Other	55 (36.7)
Comorbidity	129 (86.0)
Death	66 (44.0)
	Mean (SD)
Age (years)	63.1 (16.1)
BMI (kg/m^2^)	30.4 (6.4)
FiO_2 _(%)	64.9 (18.8)
	Median (IQR)
Hours of hospitalization before ICU admission	100 (48-220)
Mechanical breathing support (hr)	181 (120-292)
APACHE score, Mean (SD)/Median (IQR)	30.9 (4.4)/30 (28-33)
SOFA score, Mean (SD)/Median (IQR)	7.5 (2.1)/7 (6-9)

Markers regarding renal and hepatic function are presented in Table 3.

**Table 3:** Βiochemical and specific markers of renal and hepatic function ^*^Z-value and p-value from the Wilcoxon signed-rank test; INR: international normalized ratio

	Day 0		Day 4		Z^*^	P^*^
	Mean (SD)	Median (IQR)	Mean (SD)	Median (IQR)		
Bilirubin (mg/dl)	0.75 (0.70)	0.5 (0.4 ─ 0.8)	0.78 (0.72)	0.6 (0.4 ─ 0.9)	-0.91	0.363
INR	1.26 (0.30)	1.2 (1.1 ─ 1.3)	1.32 (0.88)	1.2 (1.1 ─ 1.32)	-0.48	0.629
Total protein (g/dl)	6.19 (2.92)	6 (5.4 ─ 6.3)	6.01 (0.81)	6 (5.4 ─ 6.6)	-2.57	0.010
Albumin (g/dl)	2.22 (0.71)	2.15 (1.8 ─ 2.5)	2.07 (0.72)	2 (1.7 ─ 2.3)	-5.32	<0.001
Urea (mg/dl)	61.1 (38.7)	50 (32 ─ 77)	74.5 (43.9)	60.5 (38 ─ 100)	-4.31	<0.001
Creatinine (ml/min)	0.99 (0.62)	0.8 (0.6 ─ 1.2)	0.98 (0.59)	0.9 (0.6 ─ 1.2)	-0.13	0.897
Clearance (mL/min)	89.8 (66.1)	78 (38 ─ 120)	71.8 (53.6)	60 (30 ─ 100)	-0.96	<0.001

On day 4, total protein (p=0.010), albumin (p<0.001), and creatinine clearance (p<0.001) were significantly lower compared to baseline. On the contrary, urea increased significantly on day 4 compared to baseline (p<0.001). The values of MAP, APP, and FG among the four positions are presented in Table 4.

**Table 4:** MAP, APP and FG values among by day and body position ^1^p-value for time comparison within each position; ^2^p-value for position comparison within each day; ^3^p-value for comparing the change throughout the follow-up period among body positions

	Day 1	Day 2	Day 3	F (2.148)	P^1^	F (6.894)	P^3^
	Mean (SD)	Mean (SD)	Mean (SD)				
Mean Arterial Pressure (MAP) (mmHg)							
0 degrees	91.2 (12.5)	92.5 (12.3)	92.3 (12.8)	1.44	0.241	1.47	0.209
20 degrees	89.9 (12.3)	91.5 (11.7)	91.9 (13.4)	3.29	0.040		
30 degrees	88.9 (13.2)	91.3 (11.1)	91.8 (13.3)	6.11	0.003		
45 degrees	91.1 (11.1)	92 (10.9)	93 (12.1)	3.37	0.037		
F (3, 147)	6.31	1.41	2.25				
P^2^	<0.001	0.243	0.085				
Abdominal perfusion pressure (APP) (mmHg)							
0 degrees	83.8 (13)	85 (12.5)	84.5 (13)	1.01	0.368	1.78	0.127
20 degrees	79.3 (12.8)	80.6 (12.2)	80.7 (13.7)	1.76	0.175		
30 degrees	76.5 (13.1)	78.4 (11.6)	78.8 (13.7)	4.67	0.011		
45 degrees	76.2 (11.5)	76.7 (11.4)	78.1 (12.6)	4.26	0.016		
F (3, 147)	34.75	31.56	24.29				
P^2^	<0.001	<0.001	<0.001				
Filtration Gradient (FG) (mmHg)							
0 degrees	76.2 (13.9)	77.7 (14.1)	76.7 (14.1)	1.45	0.239	1.65	0.154
20 degrees	68.7 (14.3)	69.8 (13.6)	70.2 (14.7)	1.58	0.209		
30 degrees	63.6 (14.4)	65.5 (13.8)	66 (15.1)	4.54	0.013		
45 degrees	61 (12.6)	61.6 (13)	62.6 (14.4)	1.63	0.199		
F (3, 147)	97.85	93.54	86.04				
P^2^	<0.001	<0.001	<0.001				

Out of 1800 registrations (150 patients, monitored for 3 days each and in four positions daily), IAH was reported 757 times (42.1%) and ACS 51 times (2.8%). Fourteen (9,3%) patients developed ΙΑΗ (stage 1, N=12 (8%) and stage 2, N=2 (1,3%)).

Percentages and the stage of IAH, as well as the percentages of ACS for every day and body position, are analytically described in Table 5.

**Table 5:** Percentages, stage of IAH, and percentages of ACS by day and body position. ^*^p-value for time comparison within each body position; ^#^p-value for comparison among body positions; ^†^referred only in those with intra-abdominal hypertension

			All days combined	Day 1	Day 2	Day 3		
			n (%)	n (%)	n (%)	n (%)	χ^2^ (df)	p*
Intra-abdominal hypertension (IAH)		0 degrees	36 (4.8)	10 (6.7)	12 (8)	14 (9.3)	0.73 (2)	0.696
		20 degrees	130 (17.2)	45 (30)	38 (25.3)	47 (31.3)	1.45 (2)	0.484
		30 degrees	241 (31.8)	83 (55.3)	79 (52.7)	79 (52.7)	0.29 (2)	0.867
		45 degrees	350 (46.2)	117 (78)	118 (78.7)	115 (76.7)	0.18 (2)	0.914
		χ^2^ (df)	506.25 (3)	175.87 (3)	178.94 (3)	153.17 (3)		
		P^#^	<0.001	<0.001	<0.001	<0.001		
Abdominal compartment syndrome (ACS)		0 degrees	0 (0)	0 (0)	0 (0)	0 (0)	-	-
		20 degrees	7 (13.7)	1 (0.7)	3 (2)	3 (2)	1.61 (2)	0.708
		30 degrees	15 (29.4)	4 (2.7)	4 (2.7)	7 (4.7)	1.24 (2)	0.538
		45 degrees	29 (56.9)	10 (6.7)	9 (6)	10 (6.7)	0.07 (2)	>0.999
		χ^2^ (df)	37.51 (3)	16.62 (3)	10.79 (3)	12.00 (3)		
		P^#^	<0.001	0.001	0.009	0.007		
Stage of Intra-abdominal hypertension^†^	0 degrees	1 (12-15 mmHg)	32 (88.9)	10 (100)	10 (83.3)	12 (85.7)	4.00 (2)	0.135
		2 (16-20 mmHg)	4 (11.1)	0 (0)	2 (16.7)	2 (14.3)		
		3 (21-25 mmHg)	0 (0)	0 (0)	0 (0)	0 (0)		
		4 (>25 mmHg)	0 (0)	0 (0)	0 (0)	0 (0)		
	20 degrees	1 (12-15 mmHg)	98 (75.4)	33 (73.3)	31 (81.6)	34 (72.3)	8.47 (4)	0.076
		2 (16-20 mmHg)	26 (20)	12 (26.7)	4 (10.5)	10 (21.3)		
		3 (21-25 mmHg)	6 (4.6)	0 (0)	3 (7.9)	3 (6.4)		
		4 (>25 mmHg)	0 (0)	0 (0)	0 (0)	0 (0)		
	30 degrees	1 (12-15 mmHg)	175 (72.6)	61 (73.5)	61 (77.2)	53 (67.1)	22.46 (6)	0.001
		2 (16-20 mmHg)	53 (22)	18 (21.7)	15 (19)	20 (25.3)		
		3 (21-25 mmHg)	8 (3.3)	4 (4.8)	2 (2.5)	2 (2.5)		
		4 (>25 mmHg)	5 (2.1)	0 (0)	1 (1.3)	4 (5.1)		
	45 degrees	1 (12-15 mmHg)	183 (52.3)	58 (49.6)	68 (57.6)	57 (49.6)	7.56 (6)	0.272
		2 (16-20 mmHg)	146 (41.7)	53 (45.3)	41 (34.7)	52 (45.2)		
		3 (21-25 mmHg)	11 (3.1)	3 (2.6)	5 (4.2)	3 (2.6)		
		4 (>25 mmHg)	10 (2.9)	3 (2.6)	4 (3.4)	3 (2.6)		
		χ^2^ (df)	52.33 (9)	26.61 (9)	17.99 (9)	20.84 (9)		
		P^#^	<0.001	<0.001	<0.001	<0.001		

Within each day as well as for all 3 days combined, the percentages of IAH and ACS differed significantly among the four body positions, with the lowest percentage being in the supine position (0°) and the highest at 45°. Also, no significant differences were observed in the percentages of IAH or ACS over the 3 days in any of the body positions. Moreover, within each day as well as for all 3 days combined, the stages of IAH differed significantly among the four positions. The stages of IAH during the follow-up period changed significantly only at 30° (p=0.001), while in the rest of the body positions, no significant differences were found timewise. More specifically, at 30°, the stage increased during the follow-up period.

In order to find factors significantly associated with IAH, mixed logistic regression was applied (Table 6).

**Table 6:** Mixed logistic regression results with intra-abdominal hypertension (IAH) as the dependent variable, in a stepwise method ^*^Odds Ratio (95% Confidence Interval)

	OR (95% CI)^*^	t	P
Age	0.90 (0.84-0.97)	-2.70	0.007
Gender (Women vs Men)	0.40 (0.28-0.56)	-5.26	<0.001
ΒΜΙ	1.14 (1.10-1.18)	7.56	<0.001
Creatinine (day 0)	1.83 (1.56-2.13)	7.55	<0.001
Bilirubin (Day 0)	0.80 (0.69-0.94)	-2.81	0.005
Day			
2 vs 1	0.91 (0.79-1.04)	-1.38	0.169
3 vs 1	0.95 (0.84-1.08)	-0.78	0.435
Body position			
20^o^ vs 0^o^	5.38 (4.24-6.82)	13.90	<0.001
30^o^ vs 0^o^	20.15 (15.29-26.54)	21.37	<0.001
45^o^ vs 0^o^	79.16 (55.81-112.26)	24.54	<0.001

Greater age (OR=0.90; p=0.007) and greater bilirubin (on day 0) (OR=0.80; p=0.005) were significantly associated with lower risk for IAH. Women had 60% lower risk for IAH (OR=0.40; p<0.001), while greater BMI (OR=1.14; p<0.001) and increased creatinine (on day 0) (OR=1.83; p<0) were significantly associated with a greater risk for IAH. Time-wise, the risk of IAH remained at similar levels. However, at 20o, 30o and 45o the risk of IAH was significantly greater than at 0o. In order to find factors significantly associated with ACS, mixed logistic regression was applied (Table 7).

**Table 7:** Mixed logistic regression results with abdominal compartment syndrome (ACS) as the dependent variable, in a stepwise method ^*^Odds Ratio (95% Confidence Interval)

	OR (95% CI)^*^		t	P
Gender (Women vs Men)	0.51 (0.38 ─ 0.70)		-4.28	<0.001
International Normalized Ratio (INR) (day 0)	2.96 (2.16 ─ 4.06)		6.75	<0.001
Bilirubin (Day 0)	0.58 (0.46 ─ 0.73)		-4.57	<0.001
Day				
2 vs 1	1.00 (0.87 ─ 1.16)		-0.01	0.995
3 vs 1	1.12 (0.95 ─ 1.31)		1.39	0.166
Body position				
20^o^ vs 0^o^	1.30 (1.11 ─ 1.52)		3.26	0.001
30^o^ vs 0^o^	1.70 (1.48 ─ 1.96)		7.48	<0.001
45^o^ vs 0^o^	2.55 (2.29 ─ 2.83)		17.41	<0.001

Women had 49% lower risk of ACS (OR=0.51; p<0.001), while a greater international normalized ratio (INR) (on day 0) (OR=2.96; p<0.001) was significantly associated with a greater risk of ACS. On the other hand, greater bilirubin (on day 0) (OR=0.58; p<0.001) was significantly associated with a lower risk of ACS. Time-wise, the risk of ACS remained at similar levels. However, at 20°, 30°, and 45°, the risk of ACS was significantly lower than at 0°.

Cox regression models were used to examine whether IAH or ASC were significantly associated with patients’ outcomes. Neither IAH (HR=0.64; 95% CI: 0.32-1.31; p=0.221) nor ASC (HR=1.62; 95% CI: 0.83-3.19; p=0.159) was significantly associated with survival.

## Discussion

Head of bed elevation is commonly used in critically ill ventilated patients to reduce the risk of aspiration and incidence of VAP [5]. The present study showed a significant effect of HOB elevation on the development of IAH and ACS in critically ill ventilated patients. The mean age of the sample was 63.1 years, with a predominance of men (59.3%), while the majority of patients were obese and overweight (mean BMI: 30.4 kg/m^2^). This result is consistent with similar studies in which the mean BMI was above the normal limits [6-8], while only McBeth et al. [9] found the same BMI as our study (BMI: 30 ± 13 kg/m^2^) [6,8-9].

The prevalence of IAH in the present study was nine, 3%. A systematic review found that the prevalence of IAH ranges from 30% to 49% [10]. This wide difference in prevalence may be due to the population heterogeneity of studies (surgical or medical). It should be noted that the study was carried out during the pandemic, and the cases of patients with severe pneumonia were particularly increased. In similar studies, the population was mixed [6-7,10-11] with a predominance of pathological cases. In Samimian et al.'s study, 60% were surgical patients, while in Vasquez et al., the population of the study was patients with trauma [8,12]. Previous studies have demonstrated that IAH and ACS were more common in abdominal surgeries and in serious injuries, but evidence has shown that IAH and ACS can be developed in medical patients too [13-15].

In the present study, 1.800 measurements were performed at HOB positions of 0°-20°-30°-45° above the horizontal level for three consecutive days, three times a 24-hour period. Within each day, as well as for all 3 days combined, the percentages of IAH and of ACS differed significantly among the four body positions, with the lowest percentage being at 0° and the highest at 45°. It was recorded that the higher the HOB elevation, the higher the percentage of IAH. The results of this study confirm the findings of previous studies, which demonstrate that HOB elevation is accompanied by IAP elevation [15]. McBeth et al. showed that IAP varied significantly between different HOB elevations [9,15].

Cheatham et al. conducted a multicentre study in critically ill, mixed medical and surgical patients at risk of IAH and concluded that IAP at both 15^ο^ and 30^ο^ was significantly increased compared with IAP in the supine position [11,15]. Vasquez et al. found that IAP increased progressively when the HOB was elevated (0^o^, 15^o^, 30^o^ and 45^o^) in trauma patients [8,15]. Malbrain et al. found that IAP in HOB at 45° versus in the supine position was significantly higher in patients with increased BMI [6,15]. Obesity increases abdominal fat and decreases the abdominal wall compliance, both of which contribute to elevated intra-abdominal pressure [7]. Several studies have similarly shown that patients with higher BMI are at risk of developing IAH and its complications, including impaired organ perfusion and ACS [6-8]. This highlights the need for closer surveillance of IAP in obese patients, as early recognition and timely interventions may reduce morbidity and improve outcomes.

The findings of the present study come in contradict the hypothesis that the abdominal compartment is primarily fluid in character, since the IAP would then remain constant regardless of body position. The results of the above studies, including ours, tend to agree with the consideration of Creswell et al., that abdominal contents remain a heterogeneous mix of solid, liquid, and gaseous components with the exact composition influenced by several disease processes such as paralytic ileus, visceral edema, or ascites [15].

The upright position significantly increased IAP, especially in obese patients. Also, APP and FG were significantly reduced by position changes at the time, with the lowest value being at 45^ο^ degrees. Yi et al. also reported a significant decrease in APP and FG measurements as the HOB increased, but the mean values of their sample were lower than those in our study. This finding may be explained by the fact that although the study population was mixed, surgical patients and patients with trauma were more numerous [16]. Although in the positions of 30° and 45°, the increase of IAP exceeded at the stage 2 of IAH, no patient developed compartment syndrome. According to Poiseuille’s law, a decrease in APP does not necessarily lead to a completely consistent decrease in blood flow of abdominal organs [17].

Zhou et al. found that in mechanically ventilated patients with IAH, progressive elevation of the HOB from supine to semi-recumbent position was associated with a gradual reduction in splanchnic blood flow. They also found that splanchnic blood flow was not further reduced when the HOB was elevated from 15^ο^ to 30^ο^. The researchers suggested the selection of the optimal position in patients with abdominal hypertension [1].

The reference standard for intermittent IAP measurements remains via the bladder. Creswell et al., after the comparison of pelvic intra-peritoneal pressure via intra-peritoneal catheters with intra-bladder pressure via a Foley manometer, found an excellent agreement between the two sides’ measurements [15]. However, they also suggest further research to examine the retroperitoneal compartmental pressure within the upper abdomen, which very much contains the vessels for abdominal blood supply, as well as the kidneys themselves. However, in the present study, the reduction of APP and FG might have contributed to renal function as evidenced by decreased creatinine clearance and increased creatinine and urea. Elevated creatinine reflects impaired renal function, which results from or contributes to increased IAP [3].

These findings underscore the importance of careful monitoring of abdominal pressure in obese patients and those with impaired renal function, as early recognition of IAH may guide timely interventions to prevent progression to ACS and associated morbidity. In the present study, the IAH was not significantly associated with survival or prolonged length of stay in the ICU [15]. Malbrain et al. agree with that finding, but they also found the IAH was an independent intensive care outcome predictor [18]. On the contrary, Vidal et al. found that IAH was an independent association factor at intensive care unit in mortality and a prolonged intensive care unit stay [19].

The HOB elevation in mechanically ventilated patients has become established in the daily care of critically ill patients for the prevention of VAP. On the other hand, raising the upper body is accompanied by a significant increase in IAP. A systematic review of 10 randomized controlled trials, among others, resulted in there being no statistically significant differences in clinically suspected VAP when comparing the semi-recumbent position (45^o^) with HOB elevation at 25^o^-30^o^ [4]. In conclusion, suggesting the HOB elevation minor to 30^o^ for patients with a high risk of development of IAH and ACS.

Strengths and limitations of the study

This study, according to our knowledge, is the first one with 1800 IAP measurements in mechanically ventilated critically ill patients. However, there are some limitations. The effect of different HOB elevations and of IAH on the function of abdominal organs has not been adequately studied. The absence of randomization in this cohort study introduces the possibility of selection bias, which may limit the internal validity of our findings. Measurement bias from repeated bladder instillations and inter-operator variability is likely. Another limitation is the exclusion criteria of the study (participants under the age of 18 years old, inability to change body positions in bed, and less than 24 hours of stay) that did not permit examination of patients with high risk for IAH and ACS, and this may explain the low prevalence of IAH.

## Conclusions

Elevation of the HOB increases intra-abdominal pressure, decreases abdominal perfusion pressure, and may affect the function of abdominal organs. IAP is higher when HOB is at 45° than at 30°, and it is higher at 30^o^ than at 20^o^. Patients with increased BMI have a higher risk of developing IAH. Increased IAP values at different positions above the 0^o^ from the horizontal level are not necessarily accompanied by the appearance of IAH and ACS. However, in mechanically ventilated critically ill patients with at least one risk factor for IAH, it is suggested that IAP should be measured at least once a day and limit HOB elevation to ≤20° when it is clinically feasible.
